# Verbal monitoring in Parkinson’s disease: A comparison between internal and external monitoring

**DOI:** 10.1371/journal.pone.0182159

**Published:** 2017-08-23

**Authors:** Hanna S. Gauvin, Jolien Mertens, Peter Mariën, Patrick Santens, Barbara A. Pickut, Robert J. Hartsuiker

**Affiliations:** 1 Department of Experimental Psychology, Ghent University, Ghent, Belgium; 2 School of Psychology and Counselling, Queensland University of Technology, Brisbane, Australia; 3 Clinical and Experimental Neurolinguistics, Vrije Universiteit Brussel, Brussels, Belgium; 4 Department of Neurology, ZNA-Middelheim Hospital, Antwerp, Belgium; 5 Department of Neurology, Ghent University Hospital, Ghent University, Ghent, Belgium; 6 University of Antwerp, Wilrijk, Belgium; 7 Department of Neurology, Antwerp University Hospital, Edegem, Belgium; 8 Mercy Health Saint Mary's Hauenstein Neurosciences, Grand Rapids, Michigan, United States of America; 9 Michigan State University, College of Human Medicine, Department of Translational Science and Molecular Medicine, Grand Rapids, Michigan, United States of America; University of Akron, UNITED STATES

## Abstract

Patients with Parkinson’s disease (PD) display a variety of impairments in motor and non-motor language processes; speech is decreased on motor aspects such as amplitude, prosody and speed and on linguistic aspects including grammar and fluency. Here we investigated whether verbal monitoring is impaired and what the relative contributions of the internal and external monitoring route are on verbal monitoring in patients with PD relative to controls. Furthermore, the data were used to investigate whether internal monitoring performance could be predicted by internal speech perception tasks, as perception based monitoring theories assume. Performance of 18 patients with Parkinson’s disease was measured on two cognitive performance tasks and a battery of 11 linguistic tasks, including tasks that measured performance on internal and external monitoring. Results were compared with those of 16 age-matched healthy controls. PD patients and controls generally performed similarly on the linguistic and monitoring measures. However, we observed qualitative differences in the effects of noise masking on monitoring and disfluencies and in the extent to which the linguistic tasks predicted monitoring behavior. We suggest that the patients differ from healthy subjects in their recruitment of monitoring channels.

## Introduction

During speech production, verbal output is constantly monitored for errors. Most theories assume that there are two routes for speech monitoring; an internal and an external route. The external route monitors speech after articulation, via perception. The internal route monitors speech before articulation. Previous research in verbal monitoring has shown alterations in verbal monitoring behavior in patients with brain damage [1,2,3]. More specifically, aphasic patients show a greater reliance on the internal monitoring route for self-monitoring, compared to healthy subjects. The current paper investigates whether verbal monitoring deficits are observed in another group of patients, namely in patients with Parkinson’s disease (PD), a group that is characterized not only by motor control problems but also by language and executive function deficits all contributing to a disruption in communication skills.

The exact mechanisms of verbal monitoring are under debate. Theories of internal verbal monitoring can be roughly divided into two classes: perception-based and production-based monitoring theories. The perception-based monitoring theory (perceptual loop theory [4,5,6,7]) assumes that internal monitoring takes place via an internal loop that allows the speech plan, the phonemic representation, to be sent to the speech perception system. Within the speech perception system internal and external speech undergo the same monitoring process; the perceived speech is compared to the communicative intention by a central monitor. Thus monitoring internal speech, monitoring one’s own external speech, and monitoring someone else’s speech, all depend on the same perception mechanism. Production-based verbal monitoring theories (e.g., conflict monitoring theory [8], the hierarchical state feedback control model [9], and DIVA model [10,11]) differ with respect to how exactly an error is detected, but all crucially assume that internal verbal monitoring can take place independently of the speech perception system.

Studies of monitoring in patients with acquired or progressive brain diseases has provided important insights into verbal monitoring and the relationship between verbal monitoring during production and comprehension. First of all, intact comprehension and intact other-monitoring are not sufficient for correct self-monitoring [3, 12, 13, 14, 15, 16]. For instance, Marshall et al. [12] reported a patient with very poor comprehension of auditory materials who nevertheless frequently detected and attempted to repair her speech errors. Second, studies with patients with a variety of etiologically different neurological disorders (e.g. Alzheimer's disease, PD, or stroke) have demonstrated that self-monitoring and other-monitoring can be selectively impaired at the semantic and phonological level. For instance, Oomen et al. [3] reported a patient who demonstrated impaired monitoring for phonological errors in speech production, but no such deficit in the understanding of another speaker. An independent relationship between self and other monitoring is unexpected on the basis of the perception based monitoring theories of verbal monitoring. A third interesting finding is that patients use their monitoring channels differently in comparison to healthy controls. In several studies, patients with Broca’s aphasia have been found to strongly rely on the internal route for speech monitoring [1, 3, 17]. Patients with Wernicke’s aphasia also strongly rely on the internal monitoring route, as suggested by the production of few repairs and many disfluencies [1]. A study investigating patients with apraxia of speech found that these speakers were quick to interrupt an error, but slow to restart, suggesting that internal monitoring was relatively intact but that planning a repair was effortful [2]. Oomen et al. [3] suggested two explanations for the finding that brain damaged patients rely more on the internal monitoring route for verbal monitoring. First, the patient has a monitoring deficit in the external monitoring route and therefore must rely on internal verbal monitoring. Second, the patient suffers from an attentional deficit and can therefore only monitor one channel. As the internal monitoring process takes place before external monitoring, it is plausible that attention is directed towards the earlier internal monitoring process.

Research on language processing in patients with PD has uncovered an array of problems. Some of the observed problems represent a peripheral deficit due to motor impairments, such as articulatory imprecision and poor voice control in speech production. Speech rate can be affected in several ways. Normal speech rate is observed in mild cases of PD [18], but in more severe cases speech rate may be increased (tachylalia) [19] or decreased (bradylalia) [20, 21]. Fastination may be observed in oral output when speech rate increases during production to a rate at which intelligibility is severely affected [22].

Patients with PD show deficits in verb inflection and generation [23, 24, 25, 26] and may present disrupted semantic processing [27, 28, 29, 30, 31]. Grammatical processing deficits are observed in both production and comprehension; short and grammatically simple sentences are produced and there is difficulty in understanding long or syntactically complex sentences [32–41]. Difficulty in understanding metaphoric meaning and distinguishing between jokes and lies has also been observed [42,43].

Studies on the production of single words suggest that production processes in PD are relatively intact, but that there may be some problems in the control of these processes. Colman and Bastiaanse [44] performed a review of single word production in PD patients. The main findings of the review are as follows: verb and noun production tasks have shown impaired performance, with the former being more troublesome. For noun production a distinction is made between semantic and phonemic fluency, with the semantic fluency being most consistently impaired. Henry and Crawford [45] published a meta-analysis of 68 studies (4644 patients in total) on verbal fluency in patients with Alzheimzer’s disease. In general, phonemic and semantic fluency performance was decreased, with a biggest decrease in semantic fluency, but neither qualified as a deficit when verbal intelligence and motor speed were taken into account. Auriacombe et al. [46] investigated semantic and phonological fluency in verbal and non-verbal production tasks in patients with PD. The results of this study indicated that semantic memory itself is not impaired, but rather that the patients suffer from a recall deficit. Studies of word production support the hypothesis that executive functioning is the primary observed deficit in PD patients. As word complexity increases, word retrieval difficulties arise; e.g. object naming is performed better than the more demanding action naming [47, 48], verb complexity decreases performance on verb production [49] and fluency tasks show a decreased performance after task switching [45]. Verb fluency scores discriminate between demented, non-demented PD patients, and healthy controls, while letter or category fluency scores did not [50, 51].

Only few studies investigated whether verbal monitoring is impaired in PD. McNamara at al. [14] compared verbal monitoring behavior between patients with PD, Alzheimer’s disease and healthy controls. In this study participants’ uncorrected and repaired errors were measured during the description of the Cookie Theft Picture [52]. Twenty-two patients with a mild to moderate stage of PD (stage II or III on the Hoehn-Yahr scale) were included in this study. McNamara et al. [14] found that patients with PD only repaired around 25% of their total errors, while healthy subjects repaired between 72–92% of their errors. This finding led the authors to conclude that patients with PD have an inability to correct their output errors. However, this study suffers from several important shortcomings. First, the conclusions are based on a very short description task (with a duration of approximately 2 minutes and a total error rate of 2 per patient). Second, the scoring criteria (e.g., for distinguishing between repairs and reformulations) were not fully transparent. Third, the paper provides no information with regard to the medication used by the patients. Interestingly a further study suggested that PD patients are not always aware of their monitoring deficits. Specifically, McNamara and Durso [53] found impaired pragmatic communication skills in PD patients. Patients overestimated their own ability and were unaware of the extent of their problems.

The aim of the current study is three-fold. The first goal is to investigate if verbal monitoring is impaired in patients with PD as suggested by McNamara et al. [14]. The second goal is to investigate if verbal self-monitoring is more reliant on the internal monitoring route as compared to healthy controls, similar to the focus on internal monitoring observed in aphasic patients. The third goal is to investigate if internal and external monitoring can be predicted by measures of internal and external speech processing, respectively. To test these hypotheses, performance is measured on a large number of language tasks, targeted at several subcomponents of language comprehension and perception. A number of monitoring measures were collected in tasks that were specifically designed to investigate the contributions of the different monitoring channels. Specifically, we manipulated the availability of auditory feedback so that monitoring could either use only internal monitoring (feedback blocked by noise masking) or a combination of internal and external monitoring (normal auditory feedback). We used a regression approach to test whether monitoring performance in each feedback conditions is predicted by our measures of perception and production.

## Materials and methods

### Participants

In this study 21 native Dutch-speaking patients with PD and 21 age-matched controls were tested via a series of language tasks. PD patients were contacted beforehand to coordinate the experiment with their regular examination for adjustments of their medication scheme at Ghent University Hospital, or were recruited ad hoc during their regular follow-up examinations at ZNA Middelheim. All patients were treated with levodopa, combined with various schemes of dopamine receptor agonists and amantadine, of which an overview is presented in Table 1. These medication schemes were all individualized resulting in highly variable dose regimens, as is characteristic in PD. Of the tested patient sample, three patients had implanted deep brain stimulation device of the subthalamic nucleus.

**Table 1:** Mean (SD) age, length of Parkinson, medication usage and score on the Hoehn Yahr scale.

	Age	Length Parkinson (years)	Medication calculated as Levodopa equivalent	Hoehn Yahr Scale*
Mean	65.7 (8.5)	10.5 (6.1)	650 (298.5)	2.40 (0.9)
Range	44–80	3–24	200–1400	1–4

* Hoehn Yahr scale measures are missing for 2 patients.

Partners of PD patients taking part in this study were recruited as control participants, as they typically match on age and socio-economic status. Age-matched controls were recruited if a partner was not available to participate in the study.

Participants who ended their participation before completing all tasks (2 patients and 2 controls) were left out of the analyses. Furthermore, one patient was discarded due to clinically significant short-term memory problems and three controls, partners of patients, were excluded; one suffered from Alzheimer’s disease, one was recovering from a stroke, and one was not a native speaker of Dutch.

The data of 18 patients with PD and the 16 age matched controls (range 43–82 years, mean 64.2 years, SD 8.33 years) were taken into analyses. All participating patients were clinically diagnosed with idiopathic PD. All diagnoses were made by the fourth (P.S.) and fifth (B.P.) author of this paper according to the Unified Parkinson’s Disease Rating Scale (UPDRS) [54]. Patients reported a mean onset of the disease of 10.5 years since onset of motor problems. For one PD patient and four of the control participants, the tasks were performed at home. For all others testing took place in a quiet room in the respective hospitals.

Both patients and healthy participants provided written informed consent before participating in the study. Ethical approval for this study was obtained from the Ghent University Ethical committee, no EC/2012/176.

### Design

Each participant performed 11 tasks during the experiment. The large majority of the tasks measures performance on several aspects of speech production and perception: confrontational naming, phonological and semantic fluency, internal phonological monitoring, internal semantic monitoring, external phonological monitoring and external semantic monitoring. The language tasks were divided into the following categories: speech production tasks (n = 2), internal speech perception tasks (n = 3), external speech perception tasks (n = 2), and verbal monitoring tasks (n = 1).

Additionally, cognitive tasks, the Edinburgh Handedness Inventory (EHI) [55] and a self-designed questionnaire targeting language history, language impairments, hearing and sight were administered. An overview of the tasks is presented in Table 2. Below, each task is explained in more detail.

**Table 2:** Overview of measurement per task and per category.

Category	Task	Measurement
Control variables	Questionnaire	-Language history and pathology -Auditory perception deficits -Visual perception deficits -Perceived onset of PD
	Edinburgh Handedness Inventory	-Handedness
Cognitive Tasks	Mini mental state examination	-Global cognitive performance
	Raven’s progressive matrices	-Non-verbal intelligence
Speech Production	Boston Naming Test	-Picture naming
	Controlled word association task	-Phonological verbal fluency -Semantic verbal fluency
Internal Speech Perception	PALPA test 27 Homophone decision	-Internal phonological monitoring
	Phoneme monitoring task	-Internal phonological / orthographical monitoring
	PALPA test 15 Visual rhyme judgement	-Internal phonological monitoring
External Speech Perception	PALPA test 15 Auditory rhyme judgement	-External speech monitoring phonology
	Network perception task	-External speech monitoring
Verbal self-monitoring	Network production task	-Internal monitoring route performance
		-External monitoring route performance
		-Speech production variables

PALPA stands for Psycholinguistic Assessments of Language Processing in Aphasia.

All computer-based tasks were programmed in E-Prime (Version 2.0) (Phoneme monitoring task, PALPA test 15 and 27) or PowerPoint (Boston Naming Test, Raven Progressive Matrices, Network Tasks) run on a portable lap-top computer. Responses were given via a Cedrus RB-x30 response box, sound was delivered via Sennheiser HD 250 over-ear headphones, and sound was recorded on a Roland R-1 mp3 recorder.

#### Cognitive performance tasks

Two cognitive performance tasks were administered: the mini mental state examination (MMSE) [56] and the Raven Matrices (Progressive or Colored version) [57].

The MMSE measures both motor and mental functioning. In a short amount of time, up to 10 minutes, the test gives a global idea of a subject's general cognitive status.

The Raven Progressive Matrices [57] is a non-verbal intelligence test based on a multiple-choice questionnaire, which is generally accepted as a good measure of fluid intelligence [58, 59]. Participants under 65 performed set B, C, and D of the Standard Progressive Matrices (SPM). For participants over 65 the Colored Progressive Matrices (CPM) are commonly used [60, 61]. Therefore, participants over 65 years of age performed set A, AB and B of the CPM. The administered colored and standard versions both contained 36 items. The matrices were presented on a computer screen, participants gave their response verbally and in a self-paced manner. Responses were transcribed by the experimenter.

#### Speech production tasks

Two tests of speech production were administered: the Boston Naming Test (BNT), and the Controlled Oral Word Association Test (COWAT). The BNT [62] is a measure of confrontational naming. Participants name 60 black and white outline drawings of objects and animals. With each item naming difficulty increases, as word frequency decreases: item 1 is ‘bed’, and the final item is ‘abacus’. The BNT is a widely used tool to assess damage in word retrieval capacities in etiologically heterogeneous adult and pediatric patients (see for instance [63] for an overview). Decreased performance was observed for both normal cognitive decline associated with age and in patients with dementia of the Alzheimer type [64, 65]. Concurrent with standard Dutch administration, the PD patients were shown all 60 pictures, starting with picture 1. No time pressure was applied, and no cues were given. In the control group, participants started the test at item 30. If an error was made before item 38, the test returned to item 29 and was continued backwards until 8 consecutive items were named correctly. Once 8 consecutive items were named correctly, naming resumed at item 39. If in the control group the pictures were interpreted incorrectly, a semantic cue was given. If after 20 seconds no response was given, a phonological cue was given. Responses were tape recorded and transcribed verbatim.

The COWAT [66] measures phonological and semantic verbal fluency. In age related cognitive decline and Alzheimer’s disease category fluency is impaired, while letter fluency remains spared [67, 68]. Performance on this task is also affected by brain damage (e.g. [69] for an overview). Participants name as many words as possible according to a given instruction (words starting with a specific letter or belonging to a specific semantic category) in 60 seconds. The current experiment used a version for which norms for Dutch speaking elderly exist [70]. Participants named as many words as possible starting with the letters N/A/K and items from the categories ‘professions’ and ‘animals’. Responses were tape recorded and transcribed verbatim.

#### Internal speech perception tasks

Three internal speech perception tasks were performed: a homophone decision task, a phoneme monitoring task and a visual rhyme judgment task. All internal speech perception tasks require the participant to internally formulate the sound representation of one or multiple words to complete the task. In essence these tasks all tap into the process of internal speech monitoring.

The homophone decision task was taken from the Dutch version of the Psycholinguistic Assessments of Language Processing in Aphasia test battery [71] (PALPA test 27). In this task two printed words are presented on a computer screen and the participant indicates via a button press whether the word is a homophone (sounds the same) or not. In total 44 word pairs are presented, of which 25% are homophone word pairs, 25% are homophone non-word pairs, 25% are non-homophone word pairs, and 25% are non-homophone non-word pairs.

The phoneme-monitoring task [72] was adjusted in this study, and built up from three tasks: a familiarization task, a naming task, and a phoneme-monitoring task. In the experimental part (the phoneme monitoring task) a phoneme and a picture are simultaneously presented and the participant has to indicate with a button press on a response box whether the presented phoneme is part of the name of the picture (green button on the right side) or not (red button on the left side). Each picture is presented twice: once with a phoneme that is part of the name, and once with a phoneme that is not part of the name. The self-paced procedure (familiarization, naming, and phoneme monitoring) was applied to blocks of four words. In total 46 items were presented, of which 50% were presented with a phoneme which was not part of name, and in the other 50% the phoneme was part of the name. Phoneme position varied between initial (1/3 of items), medial (1/3 of items) and final (1/3) of items for those items in which the phoneme was part of the name. Note that we use the term ‘phoneme’, as per [73] while in fact a grapheme is presented.

The visual rhyme judgment task was taken from the PALPA [71] (test 15). Two printed words are presented on a computer screen and the participant indicated via a button whether the words rhyme (green button) or not (red button).

#### External speech perception tasks

Two external speech perception tasks were administered; the auditory rhyme judgment task (PALPA test 15, [71]) and a perceptual network task based on the Network task for speech production of Oomen, Postma, and Kolk (2001). Basically these tasks tap into external speech monitoring, as the participants have to rely on their perception of external speech to perform these tasks. The auditory rhyme judgment task is the auditory version of the visual rhyme judgment task, introduced above. The participant hears two words spoken by a native speaker of Dutch through headphones and indicates via a button press whether the two words rhyme (green button) or not (red button).

In the network task’s perception condition (see below for production conditions) the participant is presented with a visual display of a dot moving through a network of black and white line drawings, and a simultaneous auditory presentation of a native speaker of Dutch explaining the route of the dot through a network. The participants are instructed to report perception of an error in the description by saying ‘yes’. In total 20 errors of varying nature (see S2 File for a transcript of the perception condition of the network task) were equally distributed over four networks.

#### Verbal self-monitoring tasks

As a verbal monitoring task we used the network description task of Oomen et al. [73]. During this task the participants produced full sentences and responded in a natural way to any speech errors they made. This allows measurement of naturalistic speech production and verbal monitoring behavior in the two groups. A manipulation in which the auditory verbal feedback is unavailable to the producer allows measurement of verbal monitoring behavior in the absence of external feedback. As a consequence, the producer can only rely on the internal verbal monitoring capabilities.

In the network task the participant was presented with a display in which five simple black and white drawing of everyday objects or animals are presented. The objects are connected through lines. At the sound of a beep, a red dot appears in the network and starts to move through the network. The participant’s task was to describe the trajectory of the dot through the network, making sure to name the picture, the direction of the movement, the curvature of the line (straight or curved), and the orientation of the line with respect to the other lines (left, right, or middle). The speed of the dot is determined by presenting the participants with networks with increasing speed. The fastest speed at which the participant was still able to give an adequate description was chosen for subsequent presentation. After speed selection, the participants were presented with 16 networks. During the presentation of half of these networks (n = 8), the participant heard loud white noise (89.6 dB). The loudness of the white noise was similar to that in Oomen et al. [73] (90 dB). During these trials, participants could not use their auditory feedback for verbal monitoring due to the noise masking, and therefore had to rely on internal speech for monitoring. Although we could not fully exclude the possibility that participants perceived external speech to some extent, we were certain that the perception of external speech, if any, was strongly impaired. The order of the networks and the presentation with or without noise was counterbalanced over participants. The total duration of the network description task was between 10 and 15 minutes.

Several measures were taken from the descriptions of the participants: completeness, number of errors repaired and the percentage of errors repaired. Completeness was measured by scoring production of the following aspects: the picture name, the direction of the ball, the form of the line (straight / curved) and the orientation with respect to the other lines (top / bottom / middle). Monitoring performance was measured by counting the number of errors and, perhaps more importantly, the percentage of errors that were repaired. Per error, numbers of syllables involved were reported and a differentiation between a number of error types was made. First of all, there are several types of disfluencies. The number of reformulations, which can be seen as repairs without the initial production of an error (e.g. ‘via een—naar rechts’ *via a- to the right*) were counted. The number of repetitions, in which the participant repeated the same words without adjustment (e.g. ‘naar het—naar het potlood’ *to the—to the pencil*) and the number of filled pauses, in which we included prolongations (e.g. ‘kromme lijn eueueueuh naar rechts’ *curved line uuuh to the right*), were also counted. Other error types differentiated included semantic errors ‘vaatwasser, nee, wasmachine’ (*dishwasher*, *no*, *washing machine*), phonological errors ‘trof- tros druiven’ (*a buns- a bunch of grapes*), and grammatical errors ‘naar het—de deur’ (*to the*_neuter_−*the*_common_ *door*_common_. Per error type, the total number of errors was reported, and the percentage of errors that were repaired.

All participants performed the tasks in the following order: questionnaire, MMSE, network production task, network perception task, homophone task, the auditory and visual rhyme task, and the phoneme-monitoring task, COWAT, BNT and finally the Raven Matrices.

### Data analysis

Data were analyzed using SPSS 22. Comparisons between the two groups of participants were performed with an independent samples t-test for normally distributed data, or with the Mann-Whitney test if normality could not be assumed. Within group comparisons for task performance were compared with a paired t-test or an ANOVA for normally distributed data, or with the Wilcoxon Signed-rank test if normality could not be assumed. Alpha level was .05.

To test whether verbal monitoring behavior is related to performance on any of the control variables, speech production variables, internal speech perception and external speech perception tasks a regression analysis was performed.

## Results

Study results are reported in the following order: control variables (manual preference, MMSE, non-verbal intelligence as measured by Raven Matrices), speech production variables (COWAT, BNT, production speed during network task), internal speech perception tasks (visual rhyme task, homophone task and phoneme monitoring task), external speech perception tasks (auditory rhyme task and network perception task) and finally the verbal monitoring variables (obtained from the network task performance. To compare reaction times, incorrect trials and trials that deviated more than 2 SD from the mean per condition were removed from the data set. Per task the data loss is reported.

To check whether the different tasks in each measure showed internal consistency, we computed correlations between the scores on the different tasks, which are reported at the end of each section. For this correlational analysis the scores of the two groups were used together. Only significant correlations are reported, or the absence of any significant correlations.

### Control variables

Each group contained one left-handed participant, as measured by the EHI. On the MMSE two PD participants had a score of 23, and one participant in the control group also scored 23. The other participants’ scored above 23, indicating healthy general cognitive functioning.

Results on the Raven Progressive Matrices showed no statistically significant difference between the PD and control group, indicating that our patients and control group matched on non-verbal intelligence. A comparison with the norms for Belgian elderly, as presented in the Tables A-B in S1 File, shows that both groups scores fell within the normal range. An overview of the scores on the control variables is presented in Table 3.

**Table 3:** Control variable measures.

	Patients	Controls
Age	65.72 (8.47)	64.19 (8.32)
Gender	4 female 14 male	5 female 11 male
Handedness	85.42 (36.60)	82.03 (41.82)
MMSE	26.83 (2.33)	27.75 (2.05)
Ravens Matrices	25.83 (4.96)	27.13 (4.76)

Mean (SD) score and standard deviation per group on age, gender, the Edinburgh Handedness Inventory, Mini Mental State Examination and Raven Progressive Matrices.

Age and performance on the MMSE were negatively correlated, *r*(34) = -.343, p = .047.

Braak, Rüb, Steur, Del Tredici and De Vos [74] found a correlation between the degree of PD and the score on the MMSE. This finding was replicated in the current study; Hoehn & Yahr scales correlate significantly with performance on the MMSE (*r*(15) = .526, p = .044). Furthermore for the PD group positive correlations were found between age x score on the Hoehn Yahr scale *r*(15) = .646, p = .009, age x MMSE, *r(*18*)* = .544, p = .020, and between length of Parkinson’s disease x implanted deep brain STN stimulation, *r(*17*)* = .617, p = .008.

### Speech production variables

Overall the PD patient group and healthy control group performed very similar with respect to speech production variables.

#### Controlled oral word association test

The COWAT disclosed no significant difference in verbal fluency performance between the two groups (means are reported in Table 4) for phonological fluency (*t*_(32)_ = -1.267, *p* = .214), nor for semantic fluency (*t*_(32)_ = 1.162, *p* = .254). The PD group, but also the controls, scored below the normal range (see Table C in S1 File).

**Table 4:** Mean (SD) performance of PD and control group on the speech production tasks.

	Patients		Controls	
	Phonological	Semantic	Phonological	Semantic
**COWAT (n)**	29.22 (10.65)	30.72 (7.79)	33.75 (10.12)	34.13 (9.29)
**BNT (n)**	52.17 (4.50)		51.13 (4.27)	
**Network Task**	Normal	Noise	Normal	Noise
Syllables/second	2.61 (0.64)	2.45 (0.67)	2.77 (0.59)	2.64 (0.58)
Duration	31.02 (5.21)	32.16 (5.05)	27.56 (2.06)	28.56 (1.49)

#### Boston naming test

There was no statistical difference in BNT performance between the two groups (U = 118.0, z = -.900, *p* = .368). The mean scores on this test are reported in Table 3. In the study by McNamara et al. [14] the PD patients scored 86%, and the age matched controls scored 74% correctly. Overall in our study performance on the BNT was better, especially for the control group. A comparison with normative data matched for age and gender, available in Table D in S1 File, revealed that performance of both groups were comparable with the normative data available for subjects with <10 years of education.

#### Network task production speed

For each participant the production speed was calculated. From two networks with noise (Network 9 and Network 11) and two networks without noise (Network 8 and Network 10) the number of produced syllables was divided by the duration of the description (counted from speech onset of the first word until speech offset of the final word). These networks were chosen to avoid effects of familiarization with the task and noise in the beginning and fatigue effects at the end of the task. A summary of the production speeds is provided in Table 3.

A Condition x Group repeated measures ANOVA for the production speed, as measured by the number of syllables produced per second yielded a significant main effect of condition (F(1,32) = 11.39, p = .002) with a decrease in production speed under noise masking, but no significant effect of group (F(1,32) = .73, p = .399). There was no interaction of condition x group (F(1,32) = .20, p = .655). There was an effect of condition in each group separately: production speed was significantly higher in the normal than the noise condition for the patient group (*z* = -2.604, *p* = .009, *r* = .61) and marginally significant also in the control group (*z* = -1.903, *p* = .057, *r* = .48).

A Condition x Group repeated measures ANOVA for the length of the descriptions, measured in seconds from the first word until the end of the last word, yielded a significant main effect of condition (F (1,32) = 13.35, p = .001) and a significant effect of group (F (1,32) = 7,15), p = .012) but no significant interaction with group (F (1,32) = .06, p = .814. The network descriptions under noise were significantly longer than those under the normal feedback condition for the patient group (*z* = -2.540, *p* = .011, *r* = .60) and marginally significant for the control group (*z* = -1.831, *p* = .067, *r* = .46).

Speech production measures showed significant correlations between the phonological fluency and semantic fluency, as measured by the COWAT (*r*(34) = .388, p = .023). There was also a significant correlation between performance on the BNT and the semantic fluency as measured by the COWAT (*r*(34) = .485, p = .004). Performance on the BNT further correlated with the speed of the network descriptions, both under normal feedback (*r*(34) = .460, p = .006) and under noise (*r*(34) = .519, p = .002). Production speed under noise correlated significantly with production speed under normal feedback (*r*(34) = .920, p < .001). Network description duration under noise correlated significantly with network description duration under normal feedback (*r*(34) = .924, p < .001). These correlations show reasonable internal consistency for the speech production variables.

### Internal speech perception

Internal speech perception tasks displayed a similar performance between the PD patients and healthy controls for the rhyme judgment and homophone monitoring task. On the phoneme monitoring task the PD patients showed a decreased performance. Summaries of performance on the internal speech perception tasks is provided in Table 5. Comparison with normative data of the tasks is available in the Tables E, F and H in S1 File.

**Table 5:** Speech perception task performance.

	Patients		Controls	
	Accuracy	Reaction times	Accuracy	Reaction times
**Rhyme Task Visual**				
R	0.97 (0.05)	2252 (1261.0)	0.99 (0.03)	1967 (633.0)
O	0.67 (0.24)	3268 (1327.7)	0.64 (0.30)	3020 (961.9)
NM	0.88 (0.15)	2798 (1720.5)	0.90 (0.15)	2510 (896.5)
NF	0.75 (0.33)	3074 (1871.0)	0.78 (0.36)	2680 (860.5)
**Homophone Task**				
Homophone	0.84 (0.11)	2932 (1398.9)	0.78 (0.15)	2875 (1150.7)
Non-Homophone	0.83 (0.19)	3381 (2007.5)	0.85 (0.19)	2878 (1155.6)
**Phoneme Monitoring Task**				
Initial	0.95 (0.07)	1774 (453.2)	0.97 (0.07)	1745 (337.3)
Medial	0.87 (0.12)	2169 (468.5)	0.95 (0.08)	2065 (400.0)
Final	0.86 (0.16)	2187 (594.7)	0.96 (0.05)	2068 (352.1)

Mean (SD) accuracy and mean (SD) reaction times in milliseconds on the internal speech perception tasks.

#### Rhyme judgment task visual

In the visual rhyme judgment task for the PD group 22.0% of trials was discarded after outlier removal, and of the control group 20.8% of trials was discarded.

In the experiment the participants were presented with four types of stimuli which differed with respect to orthographic transparency and rhyme. Of the rhyming word pairs 50% had a transparent rhyming orthography (denoted as ‘R’ in the PALPA test battery) (‘bank’ *bank*–‘mank’ *limp)*, and in 50% of the cases the rhyme was orthographically opaque (O) (‘krijt’ *crayon*–‘meid’ *girl)*. Of the word pairs that did not rhyme, 50% had a different medial vowel (NM) (‘grof’ *coarse*–‘graaf’ *count*) and 50% had a different final consonant (NF) (‘pen’ *pen*–‘pek’ *pitch)*.

Accuracies showed no main effect of Group, but a main effect of Condition (F_1_ (1.95, 62.40) = 16.52, p < .001, ηρ^2^ = 0.34, F_2_ (3,56) = 52.00, p < .001, ηρ^2^ = 0.74). No significant interactions were observed. Post-hoc paired t-tests revealed that accuracies to all conditions differed significantly from each other (largest p = .002), except for the O and NF condition (t_1_(33) = 1.69, p = .100, t_2_(58) = 2.59, p = .012).

Response times showed a main effect of Group only by-items (F_1_ (1,30) = .547, p = .465, ηρ^2^ = 0.02, F_2_ (1,56) = 16.26, p < .001, ηρ^2^ = 0.23). A main effect of Condition was observed (F_1_ (1.78, 53.65) = 15.47, p < .001, ηρ^2^ = 0.34, F_2_ (3,56) = 30.03, p < .001, ηρ^2^ = 0.62). No significant interactions were observed. Post-hoc t-test revealed that words with transparent rhyming orthography were judged significantly faster compared to all other word pairs (largest p’s < .002). Responses to the non-rhyming word pairs did not differ significantly. A comparison between RTs in the O and NF condition was also non-significant (t_1_(32) = 1.287, p = .207, t_2_(56) = 4.091, p < .001), as was the comparison between the non-rhyming word pairs NF and NM (t_1_(32) = 1.944, p = .061, t_2_(56) = .634, p = .529)

Results indicate that visual similarity was an important factor in performance in this task; word pair with opaque rhyming were judged slowest and had lowest accuracies, followed by word pairs in which only the final letter was different.

A comparison with the normative data (see Table E in S1 File) showed that for all group performance on transparent rhyming word pairs are close to ceiling for all groups. Performance is decreased for word pairs with opaque orthography, although this is much more pronounced for the PD and control group than for the normative data group. On the non-rhyming word pairs, the PD and control group performance is decreased compared to controls (~10%), with the PD group showing a drop in performance for items in which the final vowel determines rhyme status, which is not observed in the other groups.

#### Homophone decision task

In the homophone decision task for the PD group 20.1% of trials was discarded, and of the control group 21.9% of trials was discarded after outlier removal.

Accuracies showed no main effect of Group, no main effect of Condition, and no significant interactions.

Response times showed no main effect of Group, no main effect of Condition, and no significant interaction. We note that the by-items analyses were significant for Group (F_1_(1,32) = .321, p = .58, ηρ^2^ = 0.01, F_2_(1,42) = 12.25, p < .001, ηρ^2^ = 0.22) and for the interaction between Group and Condition (F_1_(1,32) = 2.93, p = .096, ηρ^2^ = 0.08, F_2_(1,42) = 5.62, p = .022, ηρ^2^ = 0.12) but there was no main effect of Condition (F_1_(1,32) = 3.04, p = .09, ηρ^2^ = 0.09, F_2_(1,42) = 1.43, p = .24, ηρ^2^ = 0.03).

#### Phoneme monitoring task

In the phoneme monitoring task for the PD group 16.3% of trials was discarded, and of the control group 9.6% of trials was discarded after outlier removal.

Accuracies show a significant main effect of Group (F_1_(1,32) = 5.16, p = .030, ηρ^2^ = 0.14, F_2_(1,42) = 29.83, p>.001, ηρ^2^ = 0.42), a signi_f_icant main effect of Condition (F_1_(2,64) = 5.21, p = .008, ηρ^2^ = 0.14, F_2_(2,42) = 3.67, p = .034, ηρ^2^ = 0.15) and a significant interaction (F_1_(2,64) = 3.29, p = .044, ηρ^2^ = 0.09, F_2_(2,42) = 4.07, p = .024, ηρ^2^ = 0.16). The accuracy of responses to the initial phoneme were higher than those to the medial t_1_(33) = 3.11, p = .004, t_2_(14) = 2.51, p = .025 and final phoneme t_1_(33) = 2.36, p = .024, t_2_(14) = 2.57, p = .022. Accuracy of responses between medial and final phonemes did not differ significantly.

Response times show no main effect of Group (F_1_(1,32) = .35, p = .556, ηρ^2^ = 0.011, F_2_(2,42) = 3.98 p = .053, ηρ^2^ = 0.08), a main effect of Condition (F_1_(2,64) = 31.96, p < .001, ηρ^2^ = 0.5, F_2_(2,42) = 35.53 p < .001, ηρ^2^ = 0.63), and no significant interactions. Post-hoc testing revealed that responses to the initial phoneme were faster than those to the medial and final phoneme (all p’s < .001). Response times between medial and final phonemes did not differ significantly.

A comparison with the data of Özdemir et al. [72](see Table H in S1 File) shows that the three groups show different accuracy patterns, although percentages overall are comparable. The PD patients performed well on the initial phoneme, but accuracies droppes with increasing phoneme position. The control group shows steady accuracies over all three conditions. The ORL participants also show a decrease in accuracy with the position of the phoneme. Response times were twice as long for our study group than for the university students that ORL tested.

The internal speech tasks showed good internal consistency. Accuracy on the visual rhyme task correlated significantly with the accuracy on the homophone task *r*(34) = .487, p = .003, and with accuracy on the phoneme monitoring task (*r*(34) = .386, p = .024), and accuracy on the homophone task correlated with accuracy on the phoneme monitoring task (*r*(34) = .352, p = .041).

### External speech perception tasks

A similar performance was observed for both the PD patient group and the healthy controls in the external speech tasks, suggesting that both hearing and auditory speech processing are relatively unaffected by PD. Summaries of performance on the external speech perception tasks is provided in Table 6. Comparison with normative data of the auditory rhyme judgment task are shown in S1 File.

**Table 6:** Mean (SD) performance on the external speech perception tasks.

Rhyme Task Auditory	Patients		Controls	
	Accuracy	Reaction times	Accuracy	Reaction times
R	0.94 (0.24)	2607 (1013.6)	0.95 (0.21)	2432 (583.0)
O	0.83 (0.38)	2886 (1366.0)	0.79 (0.41)	2596(783)
M	0.84 (0.37)	2769 (1445.3)	0.88 (0.34)	2496 (601.3)
F	0.74 (0.44)	3008 (1284.5)	0.88 (0.33)	2679(570.2)
**Network Error Detection Task**				
	N	%	N	%
Semantic errors detected	11.50 (3.4)	55.63 (16.5)	11.13 (3.3)	57.50 (16.9)

#### Rhyme judgment task auditory

In the auditory rhyme judgment task, 16.1% of PD group trials were discarded, and 17.2% of the control group trials were discarded after outlier removal.

A main effect of Group was observed only in the F2 (F_1_(1,32) = .65, p = .43, ηρ^2^ = 0.65, F_2_(1,56) = 9.82, p = .003, ηρ^2^ = .15), a significant main effect of Condition was observed (F_1_(2.14, 68.3) = 5.23, p = .007, ηρ^2^ = 0.14, F_2_(3,56) = 13.00, p < .001, ηρ^2^ = .41) and a significant interaction between Group and Condition in the F2 only (F_1_(3,96) = 1.54, p = .208, ηρ^2^ = .046, F_2_(3,56) = 8.30, p < .001, ηρ^2^ = 0.31).

A post-hoc t-test revealed that responses to the orthographically transparent rhyming word pairs differed significantly from response accuracies to the opaque word pairs and NF word pairs (largest p .026). Only in the F2 analysis NM and NF differed significantly (p = .019).

Response times showed no main effect of Group (F_1_(1,32) = 1.4, p = .25, ηρ^2^ = .04, F_2_(1,56) = 43.67, p < .001, ηρ^2^ = 0.44), a main effect of Condition (F_1_(3,96) = 3.98, p = .01, ηρ^2^ = .11, F_2_(3,56) = 8.71, p = .01, ηρ^2^ = .32), and no significant interactions. Post-hoc t-test revealed that reaction times to the different conditions differed significantly (largest p < .02), apart from response between the F and O (t_1_(32) = 1.29, p = .21, t_2_(56) = 4.09, p < .001) condition and between the F and M (t_1_(32) = 1.94, p = .06, t_2_(56) = .634, p = .529) condition.

A comparison with the normative data (see Table E in S1 File) showed that the PD group performance decreased for item pairs in which the final letter determines that the pair does not rhyme, which was not observed in the control or normative group This suggests that the PD group has difficulty maintaining a correct representation in working memory. Control group performance decreased for the orthographically transparent items, which was not observed in the PD group or in the normative data, suggesting that the control group retrieves the orthography in processing the perceived speech.

#### Network perception task

In the network perception task, participants listened to the description of four networks, similar to the descriptions the participants gave in the production network task. The participants were instructed to immediately report an error if they heard one. The two groups showed no significant difference between the number of semantic errors detected (*t*_(32)_ = 0.326. *p* = .746). The small number of phonological errors that were presented (n = 3) did not allow statistical comparison. A summary of performance on the task is shown in Table 6.

The percentage of errors detected contrasts markedly with the findings of Oomen et al. [3] who reported an error detection rate of around 85% for controls and 65% for aphasic subjects. Both our groups detected just over half of the errors. Closer inspection of the network task reveals that our network task was probably rather difficult. Four of our semantic errors were replacements by an item that was semantically close (e.g., car by truck, rhino by elephant). Furthermore, two errors followed very quickly upon the previous error, possibly resulting in an attentional ‘deafness’ to detect the second one.

External speech task measurements showed good internal consistency; accuracy scores on the auditory rhyme task and accuracy scores on the network perception task correlated significantly (*r*(34) = .341, p = .048).

### Verbal monitoring variables

Previous research has shown that in healthy participants more errors are detected under normal feedback, in which case the participant can monitor both internal and external speech [75]. In the noise masked feedback condition, external speech is masked by a loud, white noise, allowing only internal speech to be monitored. Noise-masked speech production by healthy participants is associated with fewer speech errors, indicating the relative contribution of the external monitoring channel [76]. If in comparison to external monitoring, internal monitoring is impaired, we expect the noise masking to affect monitoring behavior.

Recordings of the network descriptions were transcribed, and errors were coded for type of error and whether they were repaired or not. Early interrupted errors were errors that were interrupted within the first syllable. As there is not enough information to further classify the error in terms of semantic, phonological, or grammatical mistakes, early interrupted errors were counted as a separate group that was always repaired. A summary of the data is provided in Table 7.

**Table 7:** Network task speech production errors.

	PD Patients		Controls	
	Normal	Noise	Normal	Noise
Early Interrupted Errors	44	34	40	47
Semantic	119	119	93	83
Phonological	15	17	8	7
Grammatical	17	15	16	3
Disfluencies	169	156	168	197
Total Number of Errors	364	341	325	337

Summary of the total number of errors produced in the network descriptions under normal and noise-masked feedback by the PD patients and control group.

There was no significant difference between performances of the two groups on the number of errors produced. Noise also did not significantly affect the production of the number of errors. Furthermore, no significant interactions were observed. A condition x error type x group repeated measures ANOVA demonstrated a significant main effect of error type (F (2.365, 75.69) = 76.33, p < .001). Post-hoc tests showed that the number of errors differed significantly between the different categories (all p < .001), except for the grammatical and phonological errors.

**Fig 1:**
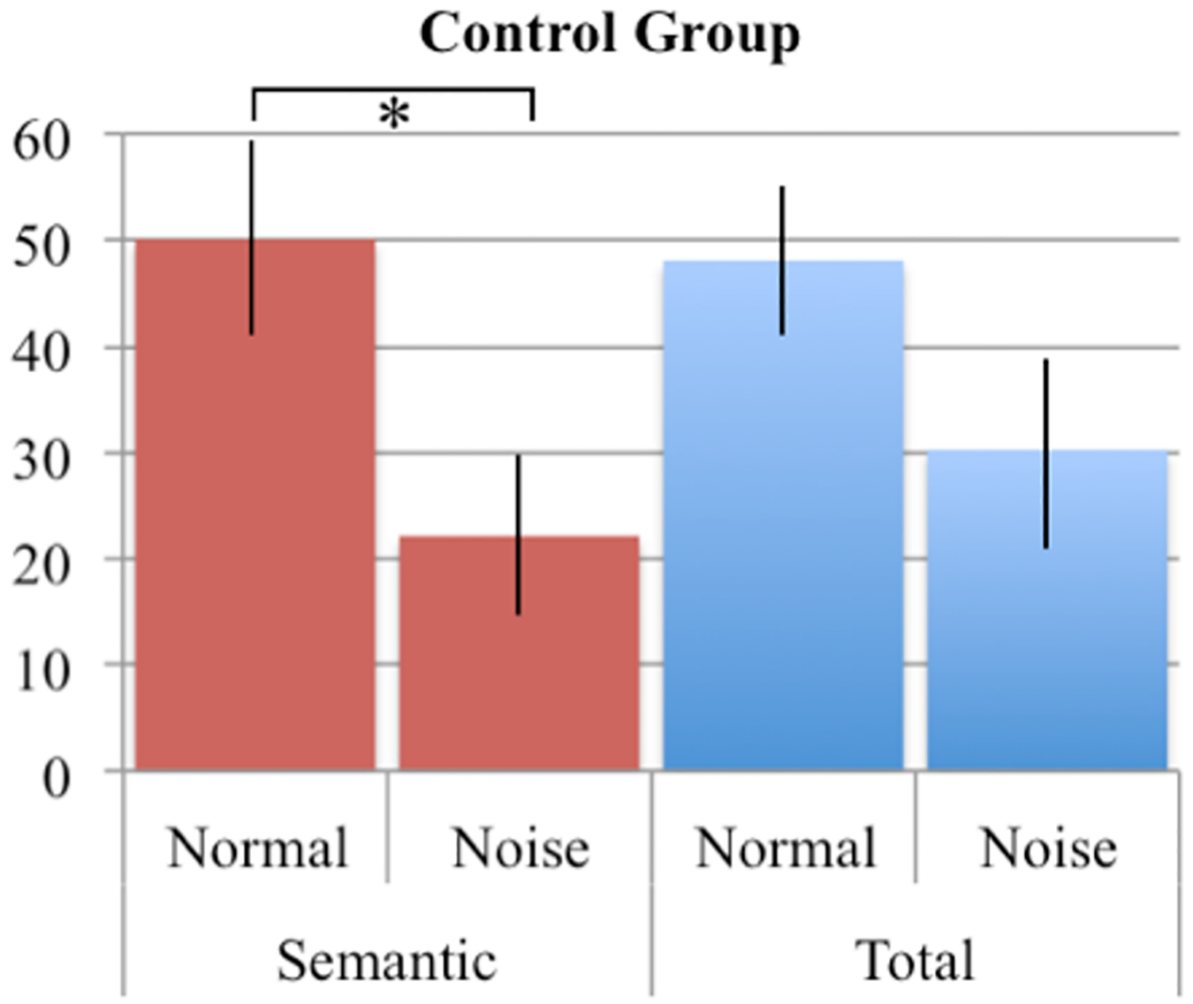
Percentage of errors repaired during production. In red are the semantic errors repaired, and in blue the total percentage of errors repaired. Error bars represent the Standard Error of the Mean.

To test the effect of noise masking on repair behavior (percentage of errors repaired) a condition x error type x group repeated measured ANOVA was performed on the data. Note that we did not take disfluencies into account here, as it is unclear whether a disfluency is the repair of an error. It might, for example, also be indicative of search behavior. Below disfluencies are extensively addressed. As the phonological errors and the grammatical errors did not have enough values for a relevant analysis, the analysis was performed on the total number of errors and on the semantic errors in the normal and noise-masked feedback condition. An overview of the repair behavior of the participants is provided in Table 8. Fig 1 shows the percentage of semantic errors and the total number of errors that were repaired. For the total percentage of errors repaired, no significant main effect or interactions were observed. For the semantic errors a significant effect of Feedback was found (F(1, 25) = 5.726, p = .025). There was a non-significant trend towards a Group x Condition interaction (F(1,25) = 2.685 p = .114). In total the two groups repaired 30.1% of the semantic errors under noise masking, while under normal feedback 46.6% of the semantic errors was repaired. A post-hoc analysis revealed a main effect of feedback on the percentage of semantic errors repaired for the control group (*t*(11)– 3.618, p = .004), but not for the PD group. However, there was no significant interaction of group x percentage of semantic errors. For the total percentage of errors repaired, no significant difference was observed between the two feedback conditions.

**Table 8:** Network task monitoring behavior.

	PD Patients			Controls
	Normal	Noise	Normal	Noise
Semantic	43.37 (38.54)	35.64 (34.14)	50.10 (35.23)	22.22 (28.93)
Phonological	50.00 (54.77)	12.50 (35.36)	69.44 (34.68)	33.33 (57.74)
Grammatical	57.43 (39.19)	35.83 (45.63)	43.33 (47.27)	100 (.00)
Total	47.71 (35.63)	31.39 (30.02)	48.08 (32.05)	30.28 (33.98)

Summary of the mean (SD) percentage of corrections per error type and standard deviations in the network descriptions by the PD patients and control group.

#### Disfluencies

Disfluencies, called prepairs by Schlenck et al. [1] or covert repairs by Levelt [4,5] have sometimes been considered repair behavior in the absence of an overt error [77]. For instance, in the verbal monitoring behavior analysis of Oomen et al. [3], disfluencies are counted as repaired errors. It is often taken to be the result of pre-articulatory, internal speech monitoring. It is therefore an interesting category of errors to investigate. Here we distinguish between reformulations, filled pauses, and repetitions. An overview of the disfluencies per type is given in Table 9.

**Table 9:** Total number of disfluencies per error type in the network descriptions.

	PD Patients		Controls	
	Normal	Noise	Normal	Noise
Reformulation	89	79	41	38
Filled Pause	49	68	89	64
Repetition	40	80	29	25
Total	178	227	159	127

A disfluencies type x condition x group ANOVA was performed on the data. No main effects were observed, but there was a significant interaction of condition x group (F(1,32) = 4.35, p = .045), with the PD patients making more disfluencies under noise masking than under normal feedback, and the controls making more disfluencies under normal feedback than under noise. Post-hoc analysis revealed no difference between the number of disfluencies between the normal and noise-masked feedback condition for both the PD and control group. An interaction was observed between disfluency type x group (F(2, 64) = 3.54, p = .035), reflecting the different error patterns: PD patients made mostly reformulations (M = 4.67), followed by repetitions (M = 3.33), and filled pauses (M = 3.25). The control group mostly produced filled pauses (M = 4.78), followed by reformulations (M = 2.47), and few repetitions (M = 1.68).

**Fig 2:**
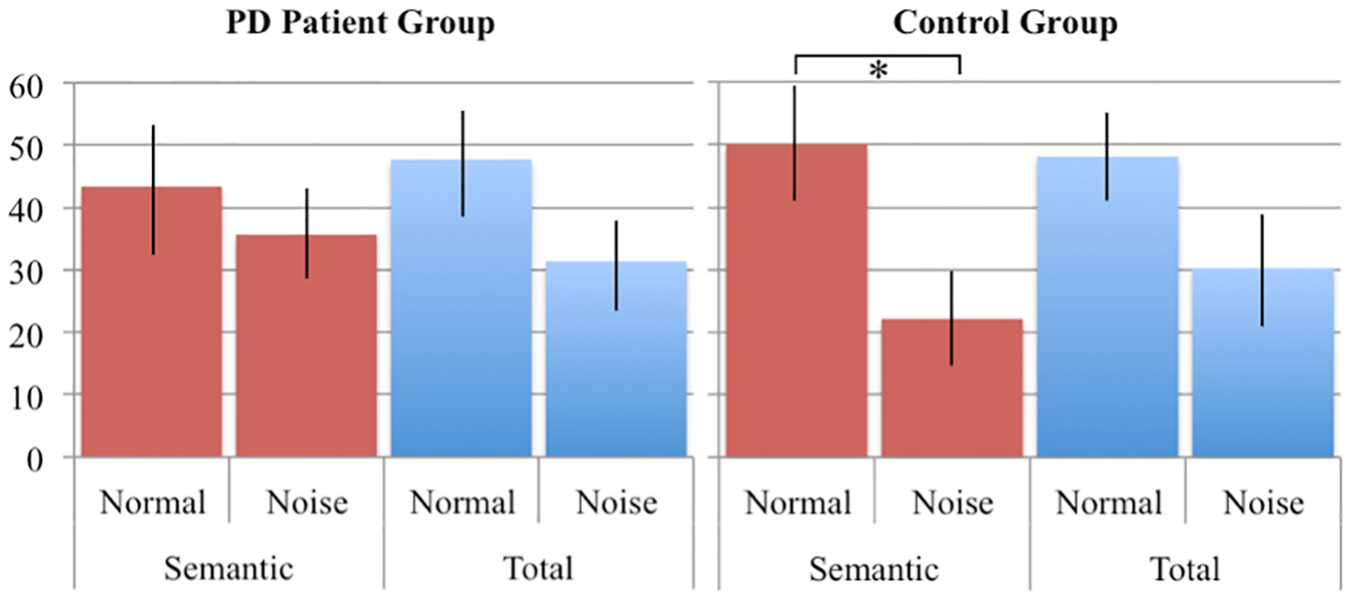
Disfluencies as a mean percentage of the total number of (overt and covert) repaired errors per person. Error bars represent the Standard Deviations.

Fig 2 shows the mean percentage of disfluencies as a percentage of the total number of overt and covert repaired errors per person, demonstrating that they count for a large proportion of the repaired errors in all conditions.

### Relationships between variables of speech processing

#### Correlations

In a study investigating 15 aphasic patients with phonological production errors, Nickels and Howard [16] found no correlation between the proportion of naming errors and performance on a comprehension task. However, a reanalysis by Roelofs [78] showed a positive correlation between performance on a homophone task and phonological self-corrections and false starts, but a negative correlation with the number of semantic errors.

Here we compared the number of semantic errors in production with the detection of semantic errors in a speech comprehension task in the PD group, analogous to Nickels and Howard [16]. We also found no significant correlation between these measures (normal feedback r(18) = -.18, noise masked feedback r(18) = -.13). In contrast to Roelofs [78], we found no significant correlation between performance on the homophone task and measures of verbal monitoring, including self-corrections and false starts. Interestingly, performance on the phoneme monitoring task showed a significant correlation with the percentage of errors repaired under normal feedback (r(18) = .47, p = .05) and under noise masked feedback (r(18) = .47, p = .05). Performance on the visual rhyme task correlated with the percentage of errors repaired under normal feedback (r(18) = .48, p = .04), but not under noise masked feedback (r(18) = .28, p = .26).

#### Regression analysis

In order to assess the relationship between verbal monitoring, internal speech and external speech, forward regression analyses were performed using the verbal monitoring performance as dependent variables and the speech task performance as independent variables. First we analyzed whether internal verbal monitoring performance could be explained by internal speech task performance. For the PD group the percentage of errors repaired under noise masking could partly be explained by performance on the phoneme-monitoring task. For the control group no such relation was found between internal verbal monitoring and internal speech processing. Next we analyzed the relationship between verbal monitoring under normal feedback and speech perception tasks. For the PD group we found that percentage of errors repaired under normal feedback conditions could be partly explained by the percentage of errors detected in the network perception task. For the control group no relation was found between external monitoring and external speech perception.

As monitoring under normal feedback consists of both internal and external monitoring, we analyzed the monitoring performance with performance on both internal and external speech tasks. For the PD group normal monitoring performance was only significantly explained by error detection in the network perception task, an external speech-processing task. For the control group no relation between normal verbal monitoring and speech task performance was found.

Normal verbal monitoring is assumed to encompass both internal verbal monitoring and additionally the monitoring, which is performed on the external speech perception. Therefore one would expect that monitoring under normal feedback could largely be explained by internal monitoring performance. We therefore entered verbal monitoring performance under normal feedback as dependent variable and verbal monitoring performance under noise-masking as independent variable, which yielded a null result. Results of the forward regression are presented in Table 10.

**Table 10:** Regression analyses of monitoring variables.

	B	SE B	β	R^2^	Sig.
Percentage of errors repaired under noise-masked feedback					
PD Group					
Constant	26.79	23.00			
Phoneme Monitoring Task Accuracy	55.02	25.57	.47	.224	.047
Control Group	/				
Percentage of errors repaired under normal feedback					
PD Group					
Constant	38.51	16.07			
Network perception task performance	3.19	1.26	.534	.286	.022
Control Group	/				

Results of the regression analysis with monitoring variables as dependent variables, and measured variables of cognitive performance, speech production measures, external speech perception, internal speech perception as independent variables.

From the regression analysis we expected to find that internal monitoring performance, as measured during noise-masked feedback, would be predicted by performance on the internal speech tasks. External monitoring behavior was expected to be predicted by external speech task performance. The results of the analysis did not completely support these predictions. The PD patients show the expected pattern of results, in which verbal monitoring under noise can be partly explained by internal speech task performance, and verbal monitoring with normal feedback can be partly explained by verbal monitoring of external speech produced by someone else. For the control group no such relationship was found. These results suggest that verbal monitoring takes place differently in healthy controls compared to patients with PD.

Furthermore, during normal speech production the speech is monitored for errors before production (the internal verbal monitoring route) and after production via the external monitoring route. Therefore, from a theoretical perspective, we would predict normal verbal monitoring to comprise of internal verbal monitoring and additionally of monitoring what is available through the external route. Importantly, verbal monitoring performance under normal speech was not explained by performance on the internal speech task, and external verbal monitoring performance variation could not be explained by internal verbal monitoring performance.

To assess the effect of PD on the variables measured in this study, a separate multiple regression analysis was performed in which task performance formed the dependent variables and the reported disease duration, the amount of medication and the score on the Hoehn & Yahr scale formed the independent variables. The dependent variables were phonological fluency, semantic fluency, BNT score, homophone task accuracy, phoneme monitoring task accuracy, visual rhyme task accuracy, auditory thyme task accuracy, errors detected during network task perception, production speed and duration of the network descriptions, the percentages and number errors produced. A summary of the significant outcomes of the regression analysis is presented in Table 11.

**Table 11:** Regression analyses of Parkinson’s disease measures.

	B	SE B	β	R^2^	Sig.
Semantic Fluency					
Constant	38.752	3.984			
Hoehn & Yahr Scale score	-3.829	1.651	-.556	.309	.039
BNT					
Constant	61.479	2.861			
Hoehn & Yahr Scale score	-3.709	1.186	-.670	.449	.009
Production speed under normal feedback					
Constant	3.962	.342			
Hoehn & Yahr Scale score	-.580	.142	-.764	.548	.001
Production speed under noise masked feedback					
Constant	3.852	.443			
Hoehn & Yahr Scale score	-.609	.184	-.691	.478	.006
Duration Description under normal feedback					
Constant	24.385	3.060			
Length PD	.729	.287	.591	.350	.026
Duration Description under noise masked feedback					
Constant	25.132	3.029			
Length PD	.744	.284	.603	.364	.022
Number of grammatical errors produced under normal feedback					
1 Constant	.846	.296			
STN Stimulation implanted	3.154	1.109	.635	.403	.015
2 Constant	-.442	.579			
STN Stimulation implanted	2.833	.939	.568		
Length PD	.135	.055	.455	.545	.005
Percentage of Semantic Errors repaired under normal feedback					
Constant	-.269	.345			
Hoehn & Yahr Scale score	.328	.133	.636	.404	.036
Number of Semantic errors detected in Network Perception Task					
Constant	15.586	1.681			
Length PD	-.362	.157	-.553	.305	.040

Results of the regression analysis with duration of PD, Medication and Hoehn & Yahr scale score as independent variables, and task performance as dependent variables.

From these regression analyses we found that speech performance measures were related to disease severity and the time since PD onset. As the disease becomes more severe, speech becomes slower, and the number for repetitions increases. Descriptions of the network task become less complete with progression of the disease. A decreased performance on the BNT with severity of PD is suggestive of word retrieval difficulties. Interestingly, of the monitoring variables, only perception of errors produced by someone else as in the Network Task Perception was related to PD progression.

## Discussion

The primary goal of this study was to investigate whether verbal monitoring is impaired in PD patients compared to healthy controls, as found by McNamara et al. [14]. We therefore investigated whether there is a greater reliance on the internal monitoring route compared to the external route, as has been observed in patients with Broca’s aphasia. And if indeed verbal monitoring is impaired in PD patients, could this be related to a functional decrease of one of the components of language processing? To answer these questions regarding verbal monitoring behavior and language processing in PD patients, language performance was investigated by means of 11 tasks. The tasks measured the following speech processing aspects: confrontational naming, phonological and semantic fluency, internal phonological monitoring, internal semantic monitoring, external phonological monitoring and external semantic monitoring. On most of these language production and comprehension tasks PD patients and controls performed at comparable levels, suggesting relatively intact lexical and phonological processing in the PD group. Similarly and in further contrast to the findings of McNamara et al. [14] there was little difference between the groups in monitoring success. However, data from the noise masking manipulation suggested qualitative differences between the groups in how they divided the labor between internal and external monitoring. This conclusion is supported by the results from regression analyses, which indicated that PD patients and controls differ in which speech processing variables predict their monitoring performance. In sum, PD patients do not have an impairment in monitoring, but they do differ from healthy controls in *how* they monitor speech.

### Aspects of language processing

Concurrent with the monitoring behavior, we found that on most language tasks the PD group performed similar to the healthy controls. An exception was the phoneme-monitoring task on which the PD patients performed less accurately than the controls. In a regression analysis, decreased language performance was predicted by the duration and severity of PD; this was true for speech production variables and to a lesser extent for tasks that tap into semantic processing.

PD patients took longer to complete the network descriptions than healthy controls. However, speech rate did not differ between the groups. Previous research investigating how speech rate is affected in PD showed some mixed results. The finding that speech rate was not different for the PD group compared to controls is in line with a study by Metter and Hanson [19], who found a normal speech rate for mild cases of PD.

The lack of differences between the PD group and control group on the vast majority of language tasks suggests that internal speech perception, external speech perception and verbal monitoring remain relatively intact in PD patients on medication. Why did we not observe language processing deficits in the PD patients, even though there is quite some evidence indicating a range of impairments? As far as we are aware, no other studies have investigated relatively simple speech processing tasks such as phoneme monitoring, homophone judgment and rhyme judgment in PD patients. However, a considerable amount of studies has dealt with fluency task performance of PD patients. Deficits in category and letter fluency tasks have been observed [46], which in our experiment remained absent in comparison with the control group (note, however, that decreased speech fluency was related to progression of PD in the regression analysis). A closer look at studies that reported a reduced fluency clarifies the issue. Ruberg and Agid [79] found reduced verbal fluency in PD patients with dementia, a markedly different population than our study group. Troyer, Moscovitch, Winocur, Leach and Freedman [80] also reported reduced verbal fluency in PD patients with dementia, but normal fluency in PD patients without dementia. Matison, Mayeux, Rosen and Fahn [81] reported reduced category fluency, which correlated significantly with the motor speech impairment (bradykinesia) of the patients. The general physical slowing is expected to reduce performance on a timed task. Gotham, Brown and Marsen [82] reported that patients show a reduced performance of verbal fluency only when 'off' levodopa, and not when ‘on’. Berg, Björnram, Hartelius, Laakso and Johnels [83] found similar phonological fluency data between cognitively intact PD patients and the control group. However, Auriacombe et al. [46] observed reduced verbal semantic fluency in a population comparable to ours. Frank, McDade and Scott [84] found impaired confrontational naming in PD patients with dementia. However, in cognitively intact PD patients, several studies found a performance similar to the healthy controls [67, 83, 85]. As noted in the introduction, findings regarding the speed of speech production have been found to go in every direction (faster, slower, no change).

A conclusion that clearly arises from the evidence reported in the literature is that the presence of dementia and the use of medication impact language processing capabilities in patients with PD. In order to obtain a clear understanding of what aspects of language processing are or are not affected in PD, these aspects should always be taken into consideration. The data gathered in this study indicate that some aspects of language processing are observed to decrease with a progression of PD. However, as long as the patient has normal cognitive functioning and is under a carefully regulated medication scheme, language production and monitoring behavior remain normal.

### Verbal monitoring in PD

Overall the monitoring behavior of the PD patients in the current study was very similar to the healthy controls, thus suggesting that verbal monitoring is not impaired in PD. This finding is in sharp contrast to the McNamara et al. [14] study. McNamara et al. [14] investigated verbal monitoring in a group of PD patients of comparable size (n = 22). These patients were highly comparable to the patients we tested with respect to the progress of the disease (Hoehn & Yahr stage II–III). It is not clear, on the other hand, whether McNamara's [14] patients had comparable medication schemes to our patients; the authors provided no information about this. In McNamara et al.'s study [14] PD patients corrected only 25% of their errors whereas healthy controls repaired 72–92% of their errors. In our study the PD patients repaired on average 47% of their speech errors under normal feedback and the healthy controls repair 48%. It is important to keep in mind though that the percentages for repairs in the McNamara study [14] were calculated from very small numbers: repair percentages were based on a sample of 3.2 errors on average for the PD group and only a single error on average for the controls. The results obtained in such small numbers of errors should be interpreted with much caution. In contrast, we had a much larger number of observations: we measured an average of 20.2 errors per PD patient, and 20.3 per healthy control in the normal feedback condition.

We also considered disfluency production under noise and normal feedback. Disfluencies are sometimes considered to reflect monitoring. This is trivially the case for reformulations, but may also hold for filled pauses and repetitions, which might reflect covert repair behavior. Under normal feedback conditions a striking difference is observed between the groups: reformulations are by far the most frequent disfluencies produced by the PD group whereas the controls predominantly produced filled pauses. A possible interpretation is that PDs are relatively slow to stop the production of a faulty utterance (consistent with a deficit in executive control). Interestingly, the disfluency pattern in patients was affected by noise in that filled pauses and repetitions increased in frequency, suggesting a shift towards internal monitoring. By contrast, disfluency production in controls was not affected by noise. This data pattern suggests qualitative differences in monitoring behavior.

#### Contributions of the internal and external monitoring route

To investigate whether PD patients relied more on internal than on external monitoring, we compared verbal monitoring performance with and without noise masking. During noise-masked speech production, only the internal monitoring route is available for monitoring, and during normal feedback the external monitoring route can be recruited additionally. If speakers strongly rely on internal monitoring, monitoring should be relatively unaffected by noise masking. But if they strongly rely on external monitoring, noise masking should impair monitoring.

Most interestingly, for the semantic errors (by far the most frequent type of error) there was a significant effect of noise masking for the control group, whereas the PD group repaired comparable number of errors in both conditions. This suggests that (at least for the detection of semantic errors), the PD patients indeed do rely more on their internal monitoring route for verbal monitoring.

Somewhat surprisingly, there was no significant Group x Feedback interaction and no effect of Feedback for the total number of errors. The fact that in the control group noise masked feedback affected semantic error monitoring suggests that different error types might be monitored by different monitoring routes. It is not very surprising that different errors are detected via different monitoring channels. For instance, Postma and Noordanus [76] showed that in healthy subjects masking auditory feedback did not affect the detection of semantic errors but decreased the detection rates of phonemic errors and disfluencies.

The finding that semantic error detection was affected by noise in controls, but not in PD patients, suggests that the two groups differ in how they detect errors of this type, with the PD patients relying less on auditory perception.

#### Is monitoring directly related to internal or external speech processing?

A set of regression analyses tested whether monitoring behavior is related to performance on any of the measured language production and comprehension components. Internal consistency of the various components was fair to good: cognitive performance tasks correlated with the age of the participant. Speech production performance, as measured with the BNT, COWAT, and the production speed of the network task, all correlated well. The internal speech perception tasks all correlated significantly with each other, as did the external speech perception tasks. Verbal monitoring, as measured by the errors produced and repaired during normal feedback and noise masked feedback, also showed a high inter-variable consistency.

Importantly, a regression analysis tested whether speech-monitoring behavior could be predicted by the performance on the internal and external speech tasks. We predicted the proportion of repaired errors under noise masking and normal feedback separately for the PD and control groups, using measures of internal speech and external speech as predictors. In the PD group, percentage errors repaired under noise was predicted by homophone monitoring accuracy, a task tapping into internal speech. The percentage of errors repaired under normal feedback, however, depended on error detection rate in the network perception task, a task clearly tapping into external speech. This suggests the patients relied on both internal and external speech, but external speech becomes a more important predictor when it is available. In contrast, error detection performance in the control group could not be predicted from performance on any of our language tasks. This was true in both the noise masking and the normal feedback conditions. Note that this is certainly not an effect of the control group which in contrast to the PD group scored at ceiling on any of our tasks (so that there would be little individual difference in task performance). In fact, as noted above, both groups had comparable results on most tasks. Thus, this analysis suggests a qualitative difference between the groups, with monitoring in patients seemingly strongly driven by internal and external speech perception, but monitoring in controls not driven by either. Thus, consistent with our conclusion from the noise masking analysis, these findings suggest qualitative differences in the recruitment of monitoring channels in the two groups.

### Theoretical implications

As indicated above, a consideration of group differences in performance on the language and monitoring tasks suggests comparable monitoring performance in PD and control groups. But the results from noise masking (at least for semantic errors) and our regression analyses indicate qualitative differences in monitoring in both groups. We suggest that these differences involve a differential recruitment of monitoring channels: therefore, monitoring performance is reduced in controls, but not in PD patients when auditory feedback is blocked. As a result, monitoring in PD patients but not in controls, can be predicted by measures of internal and external speech. The latter finding in particular, suggests that controls recruit (an) additional channel(s) compared to PD patients.

What extra channels might healthy controls recruit that PD patients have no access to? One possibility is that controls are more successful at exploiting somatosensory monitoring channels, including taction and proprioception (see Postma, 2000 for a review of possible monitoring channels, including somatosensory ones). Evidence for proprioceptive monitoring comes from Lackner and Tuller's [75] study, which showed that phonological error detection was affected by noise masking when the error was a mistake in voicing (corresponding to relatively minor sensorimotor differences) but not with mistakes in place of articulation (corresponding to a major sensorimotor difference). Given the obvious problems in PD with motor control, including their typically soft articulation, it is conceivable that somatosensory information is a less reliable channel for error detection for them as compared to controls.

In addition, our noise masking results seem to indicate that controls were in fact sensitive to masking, suggesting that the controls use auditory perception during normal speech monitoring. This finding is consistent with accounts that assume verbal monitoring to include perception speech; e.g. an auditory target or motor representation (e.g., a forward model) is compared with the actual auditory or motor percept (forward model theory, Pickering and Garrod [86, 87]; the hierarchical state feedback control model [9]; DIVA model [10,11]).

### Limitations

In the present study, effects of noise masking were limited to only one class of errors (i.e., semantic errors, the most frequent error type) for only one of our study groups (controls). There were no statistically reliable noise masking effects on error detection for the total number of errors, although there was a numeric pattern in the predicted direction. One possibility is that our noise masking procedure was not effective in blocking access to external speech. While it might be possible that the noise did not completely mask the speech produced by the participants, it is unlikely that the white noise produced at 90 dB would not have interfered with the perception of speech. Even more so as the same noise at the same volume was successful in manipulating monitoring performance in previous studies [3, 76].

The results of the regression analysis indicate that some of the aspects of language processing measured in this study (fluent speech production and semantic processing) decrease as the disease progresses. However, our sample of patients was too small to obtain group effects on these measures. By measuring a bigger sample of PD patients with a wide spread of progression of the disease, the question of whether and how these language processing aspects are affected can be investigated in more detail. Alternatively, a longitudinal study might be an interesting option to investigate how language processing in PD progresses.

While the current study did take medication intake into account, we did not explicitly test for the effect of medication. Especially since the literature suggests that medication has a big impact on language processing, a recommendation for future research into PD would be to test patients in an ‘on’ and ‘off’ medication condition.

### Summary and conclusions

Summing-up, the current study investigated whether verbal monitoring in PD patients differs from monitoring in healthy controls. To do so, we compared the performance of PD patients and controls on a battery of linguistic tasks aimed at isolating possible components shared with monitoring. We further compared the two groups on several measures of monitoring. We manipulated auditory feedback to create conditions in which speakers have to rely on internal monitoring (with the aid of noise-masking) or can use internal and external monitoring (normal auditory feedback). Finally, we predicted monitoring performance in each feedback condition from our language tasks. On the one hand, our findings suggest relatively intact language processing and monitoring on relatively simple (lexical and phonological) language tasks in this group of PD patients. On the other hand, the different effect of noise masking on repairs and disfluencies in the two groups as well as the difference in the extent to which monitoring can be predicted from the language tasks, clearly shows qualitative differences in monitoring performance in PD. We tentatively suggest that these differences involve the types of channels that speakers can recruit for monitoring, with only the healthy controls fully exploiting somatosensory feedback. Follow-up studies are needed to further test this somatosensory hypothesis, but for now one conclusion stands out: patients with PD are not worse but perform qualitatively different in self-monitoring their speech for errors.

## Supporting information

S1 File **Table A. Number of observations per percentile. Table B. Performance per participant on the Standard Progressive Matrices (SPM). Table C. Performance on the COWAT task.** Participant data are compared to normative data from Miatton et al. (2004), calculated for the age distribution within the respective groups. On the left are the data for an educational level of <12 years, and right of the / are the data for an educational level of >12 years. Significant differences between norms and group performance are indicated by *. **Table D. Scores and normative scores on BNT per participant.** Scores by participants are displayed in column 3, column 4 and 5 represent normative Mean (SD) score matched for age and gender for two levels of education. **Table E. Rhyme task performance.** Mean accuracy for PD patients, the control group and normative data. **Table F. Homophone task performance.** Mean accuracy scores PD patients, the control group and normative data. **Table G. Accuracy and reaction times in the homophone task.** W are homophone words ‘slib—slip’. NW are homophone non-words ‘mucht—mugt’. CW are non-homophone words ‘lap—lat’. CNW are non-homophone non-words ‘mub—nup’. **Table H. Performance on the phoneme monitoring task.** Mean accuracy scores for PD patients, the control group and participants in the study of Özdemir, Roelofs, and Levelt (2007).
(PDF)

S2 File Transcript perception network task. (PDF)
