# Supplementary material for: Verbal monitoring in Parkinson’s disease: A comparison between internal and external monitoring
Source: PLoS One. 2017 Aug 23;12(8):e0182159. doi: 10.1371/journal.pone.0182159 (PMC5568285; doi:10.1371/journal.pone.0182159)
Supplement: S1 File — Table A. Number of observations per percentile. Table B. Performance per participant on the Standard Progressive Matrices (SPM). Table C. Performance on the COWAT task. Participant data are compared to normative data from Miatton et al. (2004), calculated for the age distribution within the respective groups. On the left are the data for an educational level of <12 years, and right of the / are the data for an educational level of >12 years. Significant differences between norms and group performance are indicated by *. Table D. Scores and normative scores on BNT per participant. Scores by participants are displayed in column 3, column 4 and 5 represent normative Mean (SD) score matched for age and gender for two levels of education. Table E. Rhyme task performance. Mean accuracy for PD patients, the control group and normative data. Table F. Homophone task performance. Mean accuracy scores PD patients, the control group and normative data. Table G. Accuracy and reaction times in the homophone task. W are homophone words ‘slib—slip’. NW are homophone non-words ‘mucht—mugt’. CW are non-homophone words ‘lap—lat’. CNW are non-homophone non-words ‘mub—nup’. Table H. Performance on the phoneme monitoring task. Mean accuracy scores for PD patients, the control group and participants in the study of Özdemir, Roelofs, and Levelt (2007). (PDF) [file pone.0182159.s001.pdf]

S1 Appendix

Task performance comparison with normative data

Raven Matrices

Participants below 65 years of age performed set B, C and D from the standard progressive matrices. From the manual we used the normal distribution of scores to estimate their score for the full set. This estimation was used to compare with the norms for adults in Belgium to calculate the percentile of their score was. Participants older than 65 years of age performed test A, AB, B of the Colored Progressive Matrices. From the manual we used the norms for elderly people in the Netherlands to calculate at what percentile their score was. A comparison with the norms shows that the participants nicely fit with the normal range.

Table A. Number of observations per percentile.

Percentile	PD Group	Control Group
95	3	1
90	1	1
75	2	5
50	7	5
25	4	3
10	1	1

Table B. Performance per participant on the Standard Progressive Matrices (SPM).

PD Patients				Control group			
Age in years	Score	Estimation SPM total	Percentile	Age in years	Score	Estimation SPM total	Percentile
69	21		50	58	30	47	75
44	22	34	25	70	33		95
62	33	53	95	82	28		75
75	23		50	66	23		25
73	24		95	60	29	45	75
70	31		90	58	20	32	25
60	24	37	50	62	26	39	50
65	27		25	68	32		50
65	35		25	66	34		25
72	25		95	64	29	45	75
64	23	35	50	68	34		50
50	25	38	25	67	23		50
69	32		75	69	21		75
80	22		50	69	24		90
67	25		50	57	27	41	50
62	15	25	10	43	21	33	10
67	30		50				
69	28		75				
		Mean	54,7			Mean	55,9

Controlled Oral Word Association Test

Performance on the COWAT of the PD group and the control group were compared with normative data taken from Miatton et al. (2004) and tested for significance with a t-test. Normative scores of Miatton et al. (2004) were reported per age bin, which

we used for comparison with our participants. As a result, per group different normative scores were obtained. A summary of these data is presented in table 3 below. Significant differences between the group performances compared to the normative data ($p<.05$) are marked with an asterisk. As we have no educational data of the participants in this study, it is not certain which of the normative data applies to the current group. We therefore report both the results less than 12 years of education (left of the dash), and for more than 12 years of education (right of the dash). For the semantic verbal fluency in naming professions, the number of years of education yielded no differences between groups.

Performance on the COWAT task of the PD patient group differed significantly from the normative data for an educational level of more than 12 years, but not for the normative data for an educational level less than 12 years. The control group's performance on the COWAT task differed significantly from the normative data for an educational level of more than 12 years on the semantic fluency categories, and for phonological fluency of the letter A, but not from the other phonological fluency categories, nor from the normative data for an educational level of less than 12 years.

Table C. Performance on the COWAT task.

	N	A	K	Animals	Professions
PD group	8.8	8.9	11.5	18.9	11.8
Norms for PD	9.0 /*12.2	9.5 /*12.0	12.1 /*15.5	21.1 /*21.9	*15.9
Control group	11.3	9.3	13.3	19.7	14.4
Norms for Controls	9.5/12.4	9.8 /*12.1	12.4/15.8	21.8/*22.7	*16.6

Participant data are compared to normative data from Miatton et al. (2004), calculated for the age distribution within the respective groups. On the left are the data for an educational level of <12 years, and right of the / are the data for an educational level of >12 years. Significant differences between norms and group performance are indicated by *.

Boston Naming Test

Scores on the BNT were compared to the normative data for Dutch speaking Belgian elderly from Mariën, Mampaey, Vervaet, Saerens and De Deyn (1998), which were constructed on the basis of 200 native Dutch speaking elderly.

The Parkinson's Disease group showed no statistical difference with the norms for <10 years of education (z=-.261, p=.79), but a significant difference with norms for >10 years of education (z=-2.33, p=.02) The control group's performance also did not differ significantly from that of the normative group <10 years of education (z=-.647, p=.52), but did differ significantly with norms for >10 years of education (z=-2.534, p=.01).

Table D. Scores and normative scores on BNT per participant.

Parkinson's Disease Group				
Gender	Age	BNT Score	-10 yrs education	+10 years education
m	44	55	52.4 (3.67)	55.0 (3.21)
m	50	55	52.4 (3.67)	55.0 (3.21)
v	60	50	52.8 (3.70)	54.5 (2.59)

m	62	49	52.4 (3.67)	55.0 (3.21)
v	62	57	52.8 (3.70)	54.5 (2.59)
m	64	55	52.4 (3.67)	55.0 (3.21)
m	65	54	54.6 (3.69)	56.4 (1.28)
m	65	57	54.6 (3.69)	56.4 (1.28)
m	67	55	54.6 (3.69)	56.4 (1.28)
m	67	53	54.6 (3.69)	56.4 (1.28)
m	69	51	54.6 (3.69)	56.4 (1.28)
m	69	56	54.6 (3.69)	56.4 (1.28)
m	69	47	54.6 (3.69)	56.4 (1.28)
m	70	57	51.6 (5.87)	54.9 (2.81)
v	72	45	49.2 (3.94)	52.9 (4.25)
m	73	50	51.6 (5.87)	54.9 (2.81)
v	75	52	43.3 (5.45)	49.1 (6.67)
m	80	41	52.2 (2.71)	51.0 (2.65)
Total		53	52.5 (4.00)	54.8 (2.56)

Control Group

Gender	Age	BNT Score	-10 yrs education	+10 years education
v	43	56	52.8 (3.70)	54.5 (2.59)
v	57	59	52.8 (3.70)	54.5 (2.59)
v	58	51	52.8 (3.70)	54.5 (2.59)
v	58	48	52.8 (3.70)	54.5 (2.59)
m	60	51	52.4 (3.67)	55.0 (3.21)
v	62	54	52.8 (3.70)	54.5 (2.59)
m	64	56	52.4 (3.67)	55.0 (3.21)
v	66	42	49.8 (6.27)	55.0 (2.68)
v	66	53	49.8 (6.27)	55.0 (2.68)
v	67	46	49.8 (6.27)	55.0 (2.68)
v	68	50	49.8 (6.27)	55.0 (2.68)
v	68	52	49.8 (6.27)	55.0 (2.68)
m	69	52	54.6 (3.69)	56.4 (1.28)
v	69	48	49.8 (6.27)	55.0 (2.68)
v	70	53	49.2 (3.94)	52.9 (4.25)
m	82	47	52.2 (2.71)	51.0 (2.65)
Total		51	51.48 (4.61)	54.55 (2.73)

Scores by participants are displayed in column 3, column 4 and 5 represent normative Mean (SD) score matched for age and gender for two levels of education.

Rhyme task

Of the rhyming word pairs 50% has a transparent rhyming orthography (‘bank’ *bank* – ‘mank’ *limp)*, and in 50% of the cases the rhyme is orthographically opaque (O) (‘krijt’ *crayon* – ‘meid’ *girl*. Of the word pairs that don’t rhyme, 50% has a different medial vowel (NM) (‘grof’ *coarse* – ‘graaf’ *count*) and 50% has a different final consonant (NF) (‘pen’ *pen* – ‘pek’ *pitch*.

An ANOVA was conducted on the accuracy scores with modality and condition as within-subject factors, and group as between subject factors. No main effect of Group was found. A main effect of Modality ($F_1(1,32)=5.21$, $p=.03$, $\eta\rho^2=14$, $F_2\ (1,56)=8.39$ $p=.005$, $\eta\rho^2=.13$). and a main effect of Condition was found $F_1\ (3,96)=12.51$, $p<.0016$, $\eta\rho^2=28$, $F_2\ (3,56)=57.74$ $p<.001$, $\eta\rho^2=.76$) and a significant interaction between Modality and Condition $F_1\ (3,96)=9.85$, $p=.03$, $\eta\rho^2=.03$, $F_2\ (3,56)=15.94$ $p<.001$, $\eta\rho^2=.46$). Accuracies were higher for the auditory modality compared to the visual modality for both groups, and in both modalities the control group performed better than the patient group.

Compared to the normative data (table 6), both the PD patients and healthy controls scored lower on all categories in both conditions.

Table E. Rhyme task performance.

	Visual				Auditory			
	Rhyme		Non-rhyme		Rhyme		Non-rhyme	
	R	O	NM	NF	R	O	NM	NF
Patient Group	97	67	88	75	94	83	84	74
Control group	99	64	90	78	95	79	88	88
Normative data	100	90	97	98	99	94	95	93

Mean accuracy for PD patients, the control group and normative data.

Homophone task

Table F. Homophone task performance.

	Homophone		Non-homophone	
	Word	Non Word	Word	Non-Word
Patient Group	82.42	88.33	85.71	79.83
Control group	76.65	84.26	87.76	81.13
Normative data	94.67	92.86	95.67	90.29

Mean accuracy scores PD patients, the control group and normative data.

Table G. Accuracy and reaction times in the homophone task.

	Homophone		Non-Homophone	
	W	NW	CW	CNW
PD				
Accuracy	81.48(38.9)	88.1(32.5)	84.07(36.7)	80.16(40.0)
RT (s)	2587 (1379)	3314 (1919)	2919 (1619)	3704 (2554)
Controls				
Accuracy	75.42(43.1)	83.93(36.9)	87.5(33.1)	79.5(40.6)
RT (s)	2544 (1284)	3067 (1629)	2527 (1110)	3213 (1582)

W are homophone words ‘slib - slip’. NW are homophone non-words ‘mucht - mugt’. CW are non-homophone words ‘lap – lat’. CNW are non-homophone non-words ‘mub - nup’.

Phoneme monitoring task

Comparison of the data with the result from the study by Özdemir et al. (2007) (see table 9 for an overview) revealed a similar performance on accuracy between our groups and the participants of the Özdemir et al. However, RTs are much slower for

our groups. This is not surprising as our groups in contrast to the study group of Özdemir et al. (2007) consist of elderly participants.

Table H. Performance on the phoneme monitoring task.

	Accuracy			RTs		
	Initial	Medial	Final	Initial	Medial	Final
PD patients	95	87	86	1774	2169	2187
Controls	97	95	96	1745	2065	2086
O, R & L	98	93	90	846	1083	1092

Mean accuracy scores for PD patients, the control group and participants in the study of Özdemir, Roelofs, and Levelt (2007).
