# Supplementary material for: Verbal monitoring in Parkinson’s disease: A comparison between internal and external monitoring
Source: PLoS One. 2017 Aug 23;12(8):e0182159. doi: 10.1371/journal.pone.0182159 (PMC5568285; doi:10.1371/journal.pone.0182159)
Supplement: S2 File — (PDF) [file pone.0182159.s002.pdf]

S2

Transcript Perception Network Task

The scripted errors varied with respect to the origin of the error, and to have an equal distribution of errors over the four networks. Of the errors three were classified as phonological errors, all others were of semantic nature. Of the phonological errors, two were repaired. Of the semantic errors five related to the picture, four related to the shape of the line, four errors related to the location of the line or the picture, five were related to the direction of the dot movement, and one was related to the color of the dot. Of the semantic errors, one error relating to the direction of the movement was corrected.

Below, English translations of the network descriptions are provided. The correct item is presented between brackets, or when the description consists of an error and repair, the Dutch utterance is presented between brackets.

Network 1. The ball starts at the carrot in the top left [right] corner and it moves down via the left curved line to the hot air balloon. From the hot air balloon the ball returns via the right straight [curved] line to the carrot. It continues via a straight line to the left to the sparrow uhm cap [in Dutch: *mus eh muts*]. From the cap the ball goes down via the right curved line to the letter [envelope]. Then it moves left via the bottom [top] straight line to the car.

Network 2. The ball starts at the snake [snail] and travels via the left curved line to the windmill. Now it continues to the right [top] via the right curved line to the dice. From the dice it returns via the left [right] curved line. Then it travels to the right via the bottom curved line to the heart. From the ball [heart] it travels via the top [bottom] straight line to the door.

Network 3. From the umbrella the yellow [red] ball travels via the left curved line to the football above. Then it travels via the upper line to the book uhm flower [*boek eh bloem*]. From the flower the ball travels via the middle curved [straight] line back to the umbrella. From the umbrella the ball goes left via a straight line to the star. From the star it travels down via the right curved line to the car [truck].

Network 4. The ball starts at the rhinoceros [elephant] and travels via the skewed line up to the left [right] to the shirt. From the shirt the ball goes left via the bottom curved line to the hot air balloon [balloon]. From the balloon the ball goes via the straight line to the bicycle. From the bicycle the ball goes in a small [big] curve to the left [right], eh, to the bucket at the right. From the bucket the ball goes to the shirt below via a straight [curved] line.
